# Hepatitis B Screening and Prevalence Among Resettled Refugees — United States, 2006–2011

**Published:** 2015-06-05

**Authors:** Kevin C. Scott, Eboni M. Taylor, Blain Mamo, Nathaniel D. Herr, Peter J. Cronkright, Katherine Yun, Marc Altshuler, Sharmila Shetty

**Affiliations:** 1 Thomas Jefferson University, Philadelphia, Pennsylvania; 2 Division of Global Migration and Quarantine, National Center for Emerging and Zoonotic Infectious Diseases, CDC; 3 Minnesota Department of Health, St. Paul, Minnesota; 4 University of Minnesota, Minneapolis, Minnesota; 5 SUNY-Upstate Medical University, Syracuse, New York; 6 The Children’s Hospital of Philadelphia, Pennsylvania

Globally, more than two billion persons have been infected at some time with the hepatitis B virus (HBV) (1), and approximately 3.5 million refugees have chronic HBV infection (2). The endemicity of HBV varies by region (3). Because chronic hepatitis B is infectious and persons with chronic infection benefit from treatment, CDC recommends screening for HBV among all refugees who originate in countries where the prevalence of hepatitis B surface antigen (HBsAg; a marker for acute or chronic infection) is ≥2% or who are at risk for HBV because of personal characteristics such as injection drug use or household contact with an individual with HBV infection (4). Currently, almost all refugees are routinely screened for hepatitis B. However, prevalence rates of HBV infection in refugee populations recently resettled in the United States have not been determined. A multisite, retrospective study was performed to evaluate the prevalence of past HBV infection, current infection, and immunity among refugees resettled in the United States; to better characterize the burden of hepatitis B in this population; and to inform screening recommendations. The study incorporated surveillance data from a large state refugee health program and chart reviews from three U.S. sites that conduct medical screenings of refugees. The prevalence of HBV infection (current or past as determined by available titer levels) varied among refugees originating in different countries and was higher among Burmese refugees than among refugees from Bhutan or Iraq. Current or past HBV infection was also higher among adults (aged >18 years) and male refugees. These data might help inform planning by states and resettlement agencies, as well as screening decisions by health care providers.

Data for this study were collected from four sites: the Minnesota Department of Health, the State University of New York-Upstate Medical University (SUNY-Upstate), Thomas Jefferson University, and Yale-New Haven Hospital. The Minnesota Department of Health contributed surveillance data spanning 2008–2011, while each of the clinical sites provided retrospective chart review data from multiyear periods during 2006–2011, based on data availability. HBV prevalence rates among the three largest refugee groups (Bhutanese, Burmese, and Iraqi) that resettled in the United States over this period were specifically evaluated. Institutional review board approval or exemption from review was obtained at each participating site.

On the basis of the hepatitis B antibody and antigen data, the HBV status of refugees was characterized as: 1) active infection, 2) past infection without demonstration of active disease or immunity, 3) past infection with demonstration of immunity, 4) demonstration of immunity without past exposure, or 5) no evidence of past exposure or current immunity (Table 1).

Each site submitted aggregate de-identified data on HBsAg for adults (aged >18 years) and children (aged ≤18 years). Minnesota Department of Health, Thomas Jefferson University, and Yale-New Haven Hospital also submitted data on hepatitis B surface antibody (HBsAb) and hepatitis B core antibody (HBcAb), which permitted more comprehensive evaluation of current HBV status and previous exposure. In addition, both Minnesota Department of Health and Thomas Jefferson University provided information on HBV antibody titers by sex. Data from each site were compiled demographically by age, sex, and country of origin (Table 2).

Data for prevalence of HBV infection in refugees resettled in the United States were analyzed by age, sex, and country of origin using separate Z-tests for population proportions (p = 0.05) (Table 3). The 6,175 refugees who received hepatitis B screening at the four sites represent more than 95% of all refugees evaluated at each site during the periods for which data were collected. Approximately 51% of screened refugees for whom sex was reported were male, and 59% of refugees included in the study were adults (aged >18 years) (Table 2). Burmese refugees made up the largest single refugee group (39%), followed by Iraqis (13%), and Bhutanese refugees (10%). Data for other smaller refugee groups, including refugees from Eritrea, Ethiopia, Somalia, and the former Soviet Union, were combined for this study.

Data were analyzed for HBV infection status and confirmed past or current infection by country of origin, sex, and age (Table 3). Prevalence of any past HBV infection and of chronic or acute infection was significantly higher for Burmese (36.0% past; 9.4% chronic or acute) and other refugees (15.0%; 5.0%), compared with Bhutanese (5.7%; 0.9%) and Iraqi refugees (3.8%; 0.4%). Prevalence of past HBV infection among male refugees exceeded that for female refugees (23.8% and 19.5%, respectively; p<0.001).

Prevalence of any past HBV or chronic infection was higher in adults than in children (28.6% and 9.5%, respectively; p<0.001). Consistent with the implementation of routine childhood hepatitis B immunization programs for refugees from Bhutan,* Burma,† and Iraq,§ rates of immunity among refugees without confirmed past infection were significantly higher among children than adults (51.8% and 20.9%, respectively; p<0.001).

## Discussion

Refugees are almost universally screened for HBV infection on arrival in the United States (>95% of refugees evaluated at participating sites during the study period), in part, because of limited data on the prevalence of HBV infection among different refugee groups. The prevalence of past infection among Burmese, Bhutanese, and Iraqi refugees screened by participating sites at the time of U.S. resettlement was 36.0%, 5.7%, and 3.8%, respectively. Notably, the prevalence of acute or chronic HBV infection in Bhutanese refugees observed in this study more closely tracks rates seen in Nepal than in Bhutan (approximately 1% versus >5% prevalence of active HBV); most of these refugees have been living in refugee camps located in Nepal since the mid-1990s or were born in these camps (5). This highlights the importance of countries of transit as well as countries of origin to refugee health. The prevalence of chronic hepatitis B in Iraqi refugees is consistent with that reported for Iraqi refugees in San Diego, California (6).

Both active HBV infection and past infection rates were significantly higher among men than women. The reasons for this disparity are unclear, likely multifactorial, and should be explored further. Although HBV exposure among refugee children is lower than among adults, HBV infection remains an important disease among refugee children and should be evaluated in those who arrive from countries with prevalence rates ≥2%. The high rates of immunity (57.2%) among children without past infection with HBV (HBcAb negative) supports the value of overseas immunization programs, including the WHO Expanded Programme on Immunization and the CDC-Bureau of Population, Refugees, and Migration (U.S. State Department) predeparture immunization program for refugees in contributing to reduced susceptibility to, and rates of, HBV infection among refugee populations. Public health officials and health providers can use these results to target outreach efforts and screening for populations found to be at increased risk for HBV infection.

The findings in this report are subject to at least two limitations. First, although the sample is relatively large, it represents only a portion of refugees resettled in the United States during the past 5 years. Although it does contain adequate numbers of refugees from Bhutan, Burma, and Iraq to enable differentiation of the prevalence of HBV infection among these groups, refugees within each group screened at participating sites might not be representative of the group as a whole. Therefore, these results might not be generalizable to all recently resettled refugees. Second, since resettlement depends on congressional and executive actions, the refugee populations resettled in the future will likely be different from those currently being resettled in the United States. Therefore, additional studies and surveillance efforts will be required to evaluate the prevalence of HBV in these new populations.

Similar heterogeneity in disease prevalence for other diseases (both infectious and noncommunicable) has been demonstrated among other refugee populations. For example, clinically significant vitamin B12 deficiency occurs most commonly among Bhutanese refugees (7–9), while hypertension and diabetes are prevalent among Iraqi refugees (6,10). Coupled with these previous findings, data from this analysis highlight the importance of investigating prevalence and evaluating screening guidelines for other diseases based on refugee experience (i.e., both within their countries of origin and resulting from their exposures and lifestyle in transit countries). For local and state public health programs, as well as for refugee communities, such surveillance data hold the promise of making the domestic refugee medical exam more efficient and more useful by identifying the key health concerns of refugees departing from different locations, avoiding missed diagnoses while reducing unnecessary testing, and avoiding under- and over-vaccination.

What is already known on this topic?

Hepatitis B can cause acute or chronic disease and is a significant public health concern both globally and in the United States. CDC guidelines recommend hepatitis B screening in U.S.-bound refugees from countries with a prevalence of chronic hepatitis B infection ≥2%. However, many U.S. refugee health programs screen refugees universally for hepatitis B.

What is added by this report?

Evidence of active or past hepatitis B infection was found in refugee groups from Bhutan, Burma, Iraq, and other countries and varied significantly by ethnic group. Both active and past infection rates were higher among men than women.

What are the implications for public health practice?

Public health officials and health care providers can use these results to further support overseas immunization programs and to target outreach efforts to populations found to be at increased risk for HBV infection.

## Figures and Tables

**TABLE 1:** Classification of HBV status and exposure

HBV status	Exposure	HBsAb	HBsAg	HBcAb
Chronic/Acute HBV Infection	Current disease	−	+	+/−
Uncertain Current HBV Status	Past infection	−	−	+
HBV Immune	Past infection	+	−	+
	No known HBV infection	+	−	−
No HBV infection, Non-Immune	No known HBV infection	−	−	−

**Abbreviations:** HBV = hepatitis B virus; HBsAb = hepatitis B surface antibody; HBsAg = hepatitis B surface antigen; HBcAb = hepatitis B core antibody.

**TABLE 2:** Demographics of resettled refugees screened at four sites — United States, 2006–2011

	MDH		SUNY-Upstate		TJU		YNHH		All Sites	
					
Characteristic	No.	(%)	No.	(%)	No.	(%)	No.	(%)	No.	(%)
**Total screened**	**5,045**	**(100)**	**236**	**(100)**	**657**	**(100)**	**237**	**(100)**	**6,175**	**(100)**
Male*	2,558	(51)	NA	NA	348	(53)	NA	NA	2,906	(51)
Aged >18 yrs	2,920	(58)	94	(40)	479	(73)	150	(63)	3,661	(59)
**Country of origin**										
Bhutan	431	(8)	94	(40)	117	(18)	2	(1)	644	(10)
Burma	2,177	(43)	96	(41)	125	(19)	0	(0)	2,398	(39)
Iraq	406	(8)	22	(9)	253	(39)	116	(49)	797	(13)
Other†	2,031	(40)	24	(10)	162	(25)	119	(50)	2,336	(38)

**Abbreviations:** MDH = Minnesota Department of Health; NA = not available; SUNY-Upstate = State University of New York–Upstate Medical University; TJU = Thomas Jefferson University; YNHH = Yale-New Haven Hospital.

* Total for sex includes only data submitted by MDH and TJU.

† Includes all other refugee groups screened at participating sites, including those from Eritrea, Ethiopia, Somalia, and the former Soviet Union.

**TABLE 3:** Hepatitis B status in resettled refugees screened at four sites* — United States, 2006–2011

		Immune		Non-Immune		
				
Characteristic	Chronic or acute HBV infection (%)	Past infection (%)†	No evidence of infection (%)	Past infection (%)†	No evidence of infection (%)	Current or past HBV infection§ (%)
**All refugees**	**5.7**	**12.0**	**33.6**	**3.0**	**41.9**	**20.7**
Sex¶						
Male	7.3**	13.1	34.0	3.4	42.2	23.8**
Female	4.6	12.1	37.2	2.8	43.3	19.5
Unknown	1.9	4.9	10.1	2.5	31.9	9.3
Age (yrs)						
>18	7.3**	16.4	20.9	4.8	47.9	28.6**
≤18	3.4	5.7	51.8	0.4	33.3	9.5
Country of origin						
Bhutan††	0.9	4.0	22.2	0.8	57.5	5.7
Burma††	9.4**	21.8	43.5	4.8	16.7	36.0**
Iraq††	0.4	2.1	22.2	1.3	71.4	3.8
Other††,§§	5.0**	7.6	30.5	2.5	53.5	15.0**

**Abbreviation:** HBV = hepatitis B virus.

* Data from four sites: Minnesota Department of Health (MDH); State University of New York-Upstate Medical University (SUNY-Upstate); Thomas Jefferson University (TJU); Yale-New Haven Hospital (YNHH).

† Past infection is defined as a positive hepatitis B core antibody, while immunity for the purposes of this study was defined only as confirmed positive hepatitis B surface antibody.

§ Current or past HBV infection includes persons with chronic hepatitis B as well as persons with or without immunity who have documented previous exposure to hepatitis B infection.

¶ Data for sex not available from SUNY-Upstate and YNHH; totals based only on MDH and TJU data.

** Significant at p<0.001.

†† Significant (p<0.001) differences for prevalence of chronic hepatitis B and previous exposure found between the following refugee groups: Bhutan and Burma; Bhutan and other; Iraq and Burma; Iraq and other. No significant differences found between Bhutan and Iraq for prevalence of chronic hepatitis B (p = 0.184) or previous exposure (p = 0.075).

§§ Includes all other refugee groups screened at participating sites.

## Footnotes

* The Association of Medical Doctors of Asia-Nepal provides routine immunizations in Nepali refugee camps for Bhutanese refugees, including hepatitis B vaccination. Additional information available at http://www.cdc.gov/immigrantrefugeehealth/pdf/bhutanese-health-profile.pdf.

† Lack of legal recognition, limited health infrastructure, and communication difficulties have historically limited hepatitis B vaccination in Burmese refugees. However, through collaboration between CDC and the U.S. State Department’s Bureau of Population, Refugees, and Migration, U.S.-bound Burmese refugees in Malaysia and Thailand have had consistent access to pre-departure hepatitis B vaccination since 9/2013 and 12/2012, respectively. Additional information available at http://www.cdc.gov/immigrantrefugeehealth/guidelines/overseas/interventions/immunizations-schedules.html.

§ Iraq provided universal hepatitis B vaccination before recent conflicts. Hepatitis B vaccination rates among young Iraqi refugees in Syria in 2007 still exceeded 80%, and more than 50% of Iraqi refugees aged <5 years in Jordan had received vaccination. Additional information available at http://www.cdc.gov/immigrantrefugeehealth/pdf/iraqi-refugee-health-profile.pdf.

## References

[1] Te HS, Jensen DM (2010). Epidemiology of hepatitis B and C viruses: a global overview. Clin Liver Dis.

[2] Rossi C, Shrier I, Marshall L (2012). Seroprevalence of chronic hepatitis B virus infection and prior immunity in immigrants and refugees: a systematic review and meta-analysis. PLoS One.

[3] World Health Organization (2014). Global alert and response: hepatitis B.

[4] CDC (2014). Screening for hepatitis during the domestic medical examination for newly arrived refugees.

[5] Zhou YH, Liu FL, Yao ZH (2011). Comparison of HIV-, HBV-, HCV- and co-infection prevalence between Chinese and Burmese intravenous drug users of the China-Myanmar border region. PLoS One.

[6] CDC (2010). Health of resettled Iraqi refugees—San Diego County, California, October 2007–September 2009. MMWR Morb Mortal Wkly Rep.

[7] CDC (2011). Vitamin B12 deficiency in resettled Bhutanese refugees—United States, 2008–2011. MMWR Morb Mortal Wkly Rep.

[8] Benson J, Phillips C, Kay M (2013). Low vitamin B12 levels among newly-arrived refugees from Bhutan, Iran and Afghanistan: a multicentre Australian study. PLoS One.

[9] Kumar GS, Varma S, Saenger MS, Burleson M, Kohrt BA, Cantey P (2014). Noninfectious disease among the Bhutanese refugee population at a United States urban clinic. J Immigr Minor Health.

[10] Yanni EA, Naoum M, Odeh N, Han P, Coleman M, Burke H (2013). The health profile and chronic diseases comorbidities of US-bound Iraqi refugees screened by the International Organization for Migration in Jordan: 2007–2009. J Immigr Minor Health.

